# Sympathetic Discharges in intercostal and abdominal nerves

**DOI:** 10.14814/phy2.13740

**Published:** 2018-06-11

**Authors:** Tim W. Ford, Peter A. Kirkwood

**Affiliations:** ^1^ Sobell Department of Motor Neuroscience and Movement Disorders UCL Institute of Neurology Queen Square London United Kingdom

**Keywords:** gamma motoneurone discharges, Intercostal nerves, thoracic spinal cord, muscle nerve sympathetic discharges

## Abstract

There are hardly any published data on the characteristics of muscle nerve sympathetic discharges occurring in parallel with the somatic motoneurone discharges in the same nerves. Here, we take advantage of the naturally occurring respiratory activity in recordings of efferent discharges from branches of the intercostal and abdominal nerves in anesthetized cats to make this comparison. The occurrence of efferent spikes with amplitudes below that for alpha motoneurones were analyzed for cardiac modulation, using cross‐correlation between the times of the *R*‐wave of the ECG and the efferent spikes. The modulation was observed in nearly all recordings, and for all categories of nerves. It was strongest for the smallest amplitude spikes or spike‐like waveforms, which were deduced to comprise postsynaptic sympathetic discharges. New observations were: (1) that the cardiac modulation of these discharges was modest compared to most previous reports for muscle nerves; (2) that the amplitudes of the sympathetic discharges compared to those of the somatic spikes were strongly positively correlated to nerve diameter, such that, for the larger nerves, their amplitudes overlapped considerably with those of gamma motoneurone spikes. This could be explained by random summation of high rates of unit sympathetic spikes. We suggest that under some experimental circumstances this overlap could lead to considerable ambiguity in the identity of the discharges in efferent neurograms.

## Introduction

It is well recognized that the naturally occurring efferent discharges of intercostal nerves in an anesthetized or decerebrate animal may include discharges of both alpha and gamma motoneurones (Eklund et al. 1964; Sears 1964b; De Troyer et al. 2005). However, there is a third category of efferent discharges usually present in muscle nerves, those arising from the sympathetic innervation. To our knowledge, the discharges of these efferents have not been described for the intercostal nerves. Furthermore, there are few descriptions of muscle nerve sympathetic discharges recorded during ongoing somatic motoneurone activity (for refs see Boulton et al. (2016)). The intercostal nerves provide a unique set of nerve branches for investigation of sympathetic activity under such conditions. These branches, in a range of sizes, innervate various intercostal and abdominal muscles with different patterns of respiratory drive.

The nature of the intercostal nerve sympathetic discharges became a matter of interest for us during current work, in which we are reanalyzing cross‐correlation measurements of connections from expiratory bulbospinal neurones to thoracic motoneurones following spinal cord lesions (Ford and Kirkwood 1995), with a view to separately identifying connections to gamma and alpha motoneurones on the basis of efferent spike size. Separation of gamma from alpha discharges (i.e., the choice of the upper border of spike sizes for the gamma population) may be made by reference to the criteria used by Sears (1964b), but the lower size limit for the gamma population seems to be more of a problem. Inspection of recordings of the discharges at high gain revealed a dense population of very small spikes, down to the limit of discrimination from noise. It seemed likely that these discharges were those of postganglionic sympathetic efferents, but since our investigations were being made post hoc, from existing recordings, there was no possibility of identifying them by any experimental procedure. We have therefore looked for their cardiac modulation. This is a well‐known property, indeed often an identifying criterion, in other studies of muscle nerve sympathetic outflow (e.g., Boulton et al. 2014). We find that such a modulation was indeed present for these spikes for all the categories of nerves, though of a relatively modest strength. A particularly interesting feature of the presumed sympathetic discharges thus identified was that their amplitudes for the largest nerves were found to approach those of the gamma spikes.

## Material and Methods

The recordings examined came from experiments for which the main results have already been reported and were conducted according to UK legislation [Animals (Scientific Procedures) Act 1986] under Project and Personal Licences issued by the UK Home Office. The data came from 17 cats of either sex, weighing 2.5–3.7 kg. Twelve of these (vagotomized) came from the experiments reported by Saywell et al. (2007, 2011) and the other 5 (vagus nerves intact) from those reported by Road et al. (2013). The animals were anesthetized with sodium pentobarbitone (initial dose 37.5 mg kg^–1^ I.P., then I.V. as required). Neuromuscular blockade was achieved by using gallamine triethiodide (subsequent to surgery, I.V., repeated doses 24 mg as required) and the animals were artificially ventilated via a tracheal cannula with oxygen‐enriched air, to bring the end‐tidal CO_2_ fraction initially to about 4%. CO_2_ was then added to the gas mixture to raise the end‐tidal level sufficient to give a brisk respiratory discharge in the mid‐thoracic intercostal nerves (typically 6–7%). During neuromuscular blockade, anesthesia was assessed by continuous observations of the patterns of the respiratory discharges and blood pressure together with responses, if any, of both of these to a noxious pinch of the forepaw. Only minimal, transient responses were allowed before supplements (5 mg kg^–1^) of pentobarbitone were administered. The animal was supported by vertebral clamps, a clamp on the iliac crest and a plate screwed to the skull. Rectal temperature was maintained between 37°C and 38°C by a thermostatically controlled heating blanket. Mean blood pressures, measured via a femoral arterial catheter, were above 80 mmHg throughout.

### Nerve recordings

These were originally made either during spike‐triggered averaging measurements or for cross‐correlation measurements to investigate the connections from expiratory bulbospinal neurones to motoneurones or to spinal interneurones. The recordings were made from the cut central ends of selected nerves via pairs of platinum wire electrodes and with conventional amplification (filter settings 300 Hz to 3 kHz). Thoracic nerves were maintained in a single paraffin oil pool constructed from skin flaps and the lumbar nerves were recorded under petroleum jelly, most often with a piece of thin plastic film separating the electrodes from underlying muscle. These recordings were stored on magnetic tape and subsequently acquired for computer analysis via a 1401 interface and Spike2 software (Cambridge Electronic Design, Cambridge, UK). Sampling rates were around 8–10 kHz, varying according to the number of channels sampled.

In the first group of 12 cats, the recordings examined consisted of one external intercostal nerve in either T5 or T6, from one experimental run in each cat, each run being a period (no stimuli being delivered) used for intracellular recording from a motoneurone or an interneurone (1–23 min). To investigate cardiac modulation, an ECG signal was obtained from a cord dorsum recording, originally used for monitoring afferent nerve volleys during the initial testing of the motoneurones or interneurones (Saywell et al. 2011; Ford et al. 2014).

In the second group of five cats, longer runs of data (52–100 min) originally obtained for cross‐correlation measurements, were available (one run from each cat). Recordings from one external intercostal nerve (T5 or T6) and 3–6 internal intercostal nerves or nerve branches (T8–T11) from the left side of each cat were included. These branches included the following nerves on the left side of T8 and/or T9 (four cats): (1) one of the filaments of the internal intercostal nerve, which are the naturally occurring branches that leave the nerve at intervals to innervate the internal intercostal muscle layer (Sears 1964a); (2) the lateral branch of the internal intercostal nerve, which innervates external abdominal oblique; (3) the distal remainder of the internal intercostal nerve, which innervates the more distal part of the internal and parasternal intercostal muscles, transversus abdominis, and rectus abdominis (see Meehan et al. 2004 for refs). In the fifth cat, the whole internal intercostal nerves of T9 and of T11 were included, together with those of T9, T10 and T11 on the right side. In addition, in two of the animals, recordings from branches of the L1 ventral ramus were also available, including (in both animals) a branch innervating internal abdominal oblique and a distal remainder (for more details see Road et al. 2013). In only one of this group of five animals was there a cord dorsum recording that could be used to provide an ECG signal, but in the other four an ECG signal could be derived from one of the nerve recordings, in each case a nerve with a relatively modest efferent discharge.

### Analysis

This consisted firstly of visual inspection of the efferent discharge signals, concentrating on the smallest spikes viewed at high gain, secondly of the construction of cross‐correlation histograms between efferent spikes in different spike‐amplitude ranges, with *R*‐wave events as reference, over a lag range of ±0.6 sec. *R*‐wave events were obtained by triggering from the ECG signal. For more details, see Ford and Kirkwood (2018), which reports similar analyses for the alpha motoneurone spikes in the same recordings. Bin‐widths were usually 4 msec, but in a few instances, where the run length was short or the number of efferent spikes was low, a bin‐width of 10 msec was used instead, and for subsequent quantification, one of 20 msec.

## Results

### External intercostal nerves

**Figure 1:**
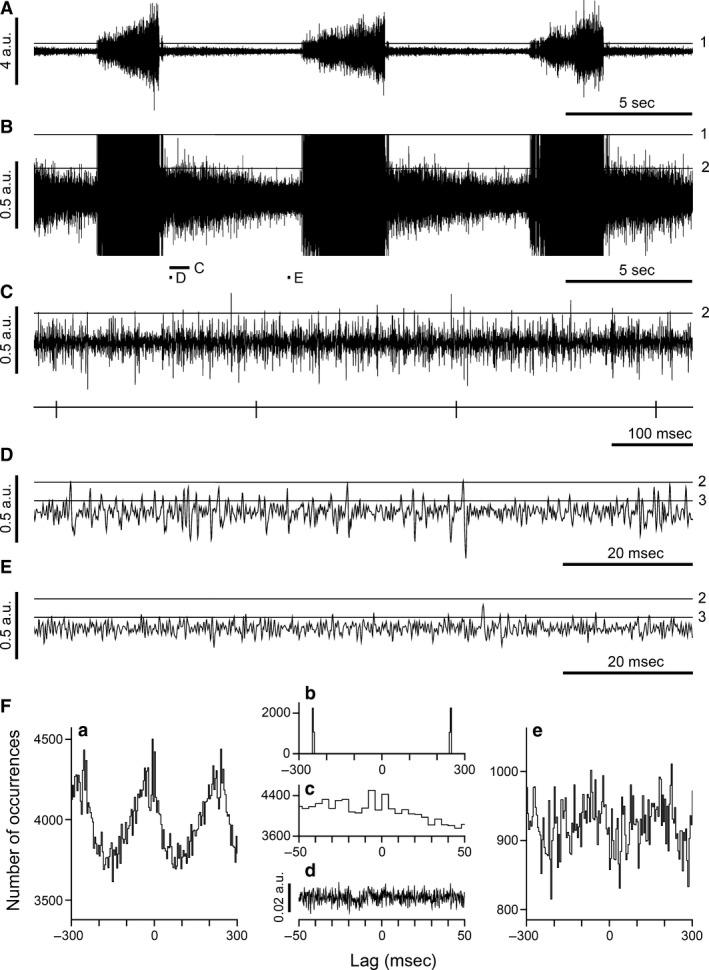
Analysis of a recording from T5 external intercostal nerve. (A–E), each panel shows an extract from the same recording with different gains and time scales. Panels (A), (B) show the same time period, but B is at 8× higher gain. (C–E), same gain as (B), but different time scales. The periods in (C–E) are expanded versions of the times indicated by the short bars in (B). Horizontal lines (1, 2, 3) indicate the spike amplitude levels separating the three ranges used for analysis. Spikes larger than level 1 were taken as alpha spikes, those between level 2 and level 1 as gamma spikes and those between level 3 and level 2 as sympathetic spikes. Bottom trace in (C), *R*‐wave events triggered from the filtered version of a cord dorsum recording (see Ford and Kirkwood (2018) for more detail). (F), cross‐correlation analyses from the data in (A–E) a, e, cross‐correlation histograms (*R*‐wave reference) for the sympathetic and gamma ranges respectively; b, auto‐correlation histogram for the *R*‐wave events (*R*‐wave period 250 msec); c, central part of the histogram shown in a, for comparison with the *R*‐wave‐triggered average of the nerve signal (d). Number of *R*‐wave events, 3309. Ordinate ranges for a, e, 0.85–1.15 m (m is the mean count calculated over 0.6 sec). Bin widths, 4 msec. The calibrations for (A–E) and for Fd are in the same arbitrary units. The same recording was illustrated in Ford and Kirkwood (2018), their Figure 1, from which Fb and Fd here are reproduced.

An example of an external intercostal nerve recording and its analysis is illustrated in Figure 1. The main bursts of spikes define inspiration, the largest spikes being derived from alpha motoneurones. Following Sears (1964b) gamma motoneurone spikes are also present and need to be differentiated from the alphas, to enable one of the comparisons we wish to make, that between the spikes of sympathetic and of gamma discharges. The criterion we used was a spike amplitude level between alpha and gamma spikes as indicated in Figure 1, a little below the amplitude of early inspiratory spikes [Fig. 1A and B, level 1]. This criterion is justified in Ford and Kirkwood (2018), where exactly the same levels were used in the analyses of the alpha discharges. Seventeen recordings of the discharges from these nerves, from either T5 or T6, were analyzed (each from one cat).

A second spike amplitude level was also required, to separate gamma spikes from sympathetic discharges, and was also indirect. It was based on the assumption, derived from the experience in this laboratory, that with elevated levels of CO_2_, the gamma discharges in external intercostal nerves cease soon after the start of expiration, that is, very soon after any postinspiratory discharge, which itself sometimes includes a few alpha spikes. We therefore set the level for separating gamma from sympathetic discharges as illustrated in the high gain recording in Figure 1 (B–E, level 2). Note that there was a high density of very small spikes (or spike‐like deflections) with amplitudes below this level, the amplitudes and frequency of these gradually decreasing as expiration progressed. There was also a subjective element in choosing this level. Although, in general, the spike trains of individual units were not separable within the gamma range, from time to time such trains were apparent, especially as the discharges declined in postinspiration. This was never the case within the lower range, which we designated as sympathetic. The spike‐like deflections in this range were not noise transients (if so, they would not have shown the modulation through expiration) and had a relatively consistent time‐course, being diphasic, starting positive going, as for the alpha and gamma deflections. Nevertheless, as one considers progressively smaller amplitudes for these spikes, one gets to the point where they merge into the baseline noise. Another arbitrary choice was therefore made in order to select spikes in this smallest range, as indicated, selecting a level by eye where most level crossings consisted of spike‐like waveforms (Fig. 1D and E, level 3). For some of the recordings, a more formal justification for the choice of levels was possible and will be described in the final section of Results.

The external intercostal nerve is a pure muscle nerve, for which a defining characteristic of its sympathetic discharges is said to be strong cardiac modulation (Jänig et al. 1983). However, such modulation was not obvious by eye in 16/17 of the recordings, including Figure 1. We therefore constructed cross‐correlation histograms between the *R*‐wave events and the events defined by the spike‐like deflections with peaks within the sympathetic range (between levels 2 and 3 in Fig. 1D and E). As illustrated in Figure 1Fa, a clear modulation with the cardiac cycle, approximately sinusoidal at the cardiac frequency (see the auto‐correlation of the *R*‐wave events in Fig. 1Fb), was almost always present.

In analyses such as these, when events were detected by level crossings close to the baseline, an obvious concern is that an ECG signal could be present in the nerve recording and might contribute to level crossings. In fact, for the 12 experiments of the first group, this was never a problem. An ECG effect was only detected in 3 of these 12 recordings, one of which (typical of these 3) was that illustrated in Figure 1. The ECG was not visible in the raw data, though it could be detected by averaging the nerve signal, triggered by the *R*‐wave events (Fig. 1Fd). The *QRS* signal detected was very small, entirely within the baseline noise in Figure 1 (note the voltage scale, the arbitrary units in Fd, being the same as shown for the raw data in A–E). Nevertheless, an effect of this signal in the histogram for the sympathetic discharges appeared as a triphasic feature, corresponding closely to the averaged ECG waveform (see expanded trace in Fc). Although clear, this was a negligible effect, and was one that was easily separable from the rest of the histogram time‐course, as was the case in the other two recordings where a similar artifact appeared. In the second group of five experiments (multiple nerve recordings, no intracellular recordings), ECG waveforms were more common and were larger. It is not known why the two series of experiments differed in this regard. Perhaps it was a result of different earthing (grounding) arrangements in the two series. Despite the larger amplitudes of the ECG waveforms and their much larger effects in the histograms, it remained possible to separate the ECG effects from the near sinusoidal component in the histograms, as is described in a later section (*Internal intercostal nerve filaments*). When this was done, all five of the histograms from the external intercostal nerves in this second group showed a near sinusoidal component, as did 11/12 from the first series.

In addition, we routinely constructed similar histograms from spikes with amplitudes in the gamma range. These also frequently demonstrated cardiac modulation, but this always, like the example in Figure 1Fe, had a lower amplitude than the histogram for the sympathetic range. In Figure 1Fe, the near sinusoidal waveform also appears noisier. This occurred partly as a result of the lower bin counts, but also because there was a minor contribution of relatively narrow peaks, representing some overlap from the alpha range (Ford and Kirkwood 2018).

### Internal intercostal nerves

**Figure 2:**
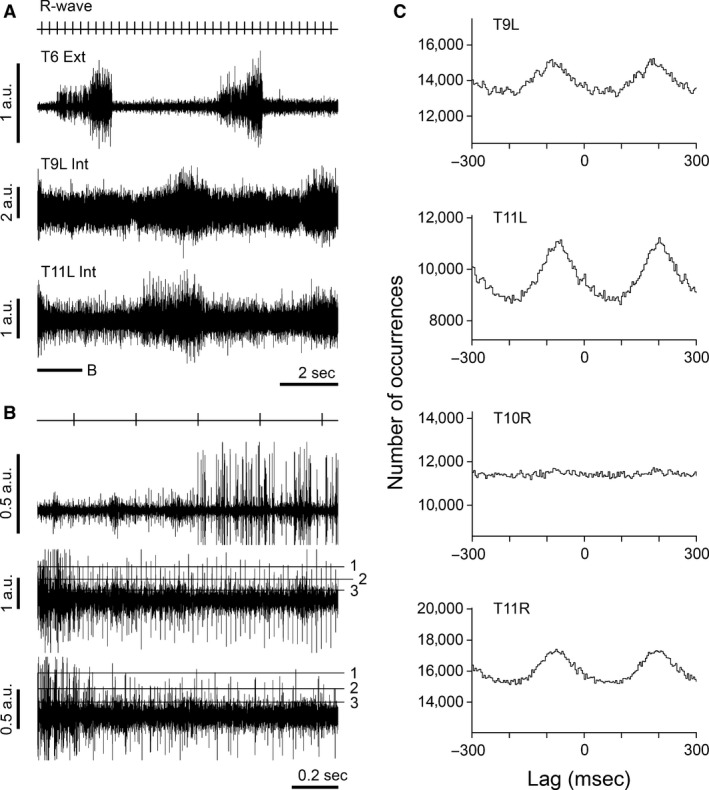
Analyses of recordings from whole internal intercostal nerves. (A) Extract from simultaneous recordings from one external (T6 Ext) and two internal (T9L Int and T11L Int) intercostal nerves from the left side together with *R*‐wave events. (B), same recordings as in (A) from the period indicated by the bar under the left of T11L Int (time scale ×8, gain ×2). Horizontal lines (1, 2, 3) show the boundaries of the spike amplitude ranges equivalent to those in Fig. 1. (C) cross‐correlation histograms for the sympathetic range (levels 3–2), as in Fig. 1Fa. Top two histograms apply to the nerve discharges illustrated in A, B. Bottom two histograms apply to simultaneous recordings from two other internal intercostal nerves on the right side of the animal. 8698 *R*‐wave events. *R*‐wave period, 271 msec. Ordinate ranges 0.8–1.2 m for all histograms. Bin widths 4 msec. The same recordings were illustrated in Ford and Kirkwood (2018), their Fig. 2, where A and B are the same as A and B here, except that different spike amplitude levels were indicated.

All of the five recordings from whole internal intercostal nerves that were analyzed came from a single experiment. Figure 2A and B shows two of these recordings, along with a simultaneous recording from an external intercostal nerve, whose main burst of discharge defined inspiration. As in Kirkwood (1995), four out of five of these recordings showed two bursts of discharges, one in expiration, another (rather weaker) in inspiration, together with an apparent tonic background, assumed to include tonic gamma discharges. The spike amplitude levels for the alpha/gamma border were assigned as described by Ford and Kirkwood (2018). Setting the gamma/sympathetic border in this animal was then helped by the fact that three of the internal intercostal nerves (including the two illustrated) and the external intercostal nerve all showed cardiac modulation of the sympathetic discharges, clearly visible in the raw data (Fig. 2B).

Cross‐correlation histograms were constructed for each nerve between the *R*‐wave marker and the two spike amplitude ranges, as previously. Each of the four nerves showed cardiac modulation for the sympathetic range, though for one of them the amplitude of this was very small (Fig. 2C). As was the case for the external intercostal nerves, the histograms for the gamma ranges (not illustrated) showed lower amplitudes of modulation than those for the sympathetic ranges. The fifth internal intercostal nerve recording in this animal (T9 right, not illustrated) showed a tonic discharge with only a very modest respiratory discharge and probably no alpha spikes. It was not considered further.

### Lateral branch

**Figure 3:**
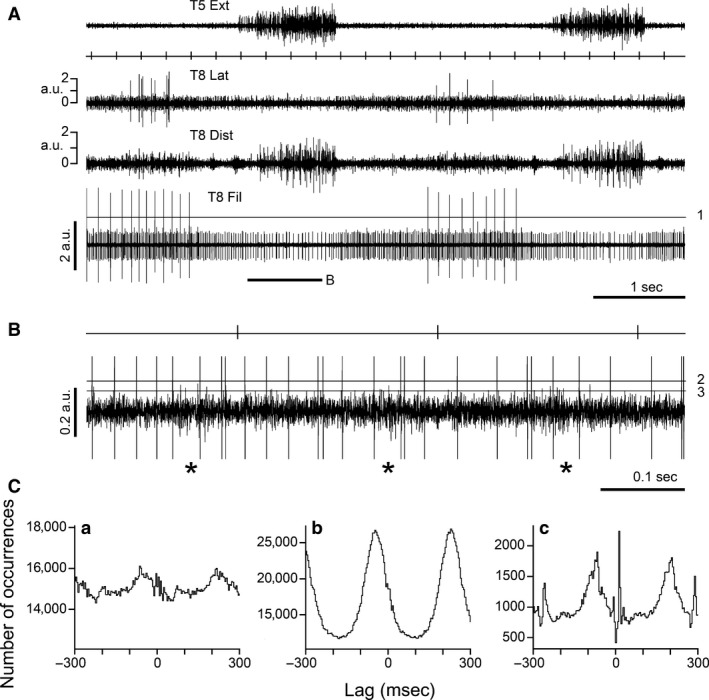
Analyses of recordings from branches of internal intercostal nerve. (A) simultaneous recordings, traces from above: T5 external intercostal nerve (T5 Ext); *R*‐wave events; lateral branch of T8 internal intercostal nerve (T8 Lat); distal remainder of T8 internal intercostal nerve (T8 Dist); T8 internal intercostal nerve filament (T8 Fil). Spike amplitude range boundaries: T8 Lat, 0.73, 0.28, 0.18; T8 Dist, 0.73, 0.38, 0.16 (a.u. on the scales illustrated). (B) extract from the filament recording (as indicated below filament trace in A) together with *R*‐wave marker (time scale ×8, gain ×10). Boundaries for spike amplitude ranges indicated by lines 1–3, as in Figure 1. Note cardiac‐related bursts of small spikes in the 4th trace, particularly in late expiration and early inspiration, and in B (*). (C) cross‐correlation histograms for the sympathetic ranges for each of the three internal intercostal nerve branches illustrated above: a, T8 Lat; b, T8 Dist; c, T8 Fil. 14647 *R*‐wave events. *R*‐wave period, 275 msec. Ordinate ranges for histograms: a, 0.8–1.2 m; b, 0.6–1.6 m; c, 0.3–2.2 m;. Bin widths 4 msec. Some of the same recordings here were illustrated in Ford and Kirkwood (2018), their Figure 3, where the first 4 traces in A here also appeared.

Recordings from six of these branches of the internal intercostal nerve (from four cats) were analyzed. These branches innervate external abdominal oblique muscle and have a cutaneous component. Note that in the example shown in Figure 3A (third trace), as in another 3 of these recordings, the alpha and gamma spikes were readily distinguishable, as in the original illustrations for the filament discharges in Sears (1964b). Also, in this particular recording, the gamma discharges were readily distinguished from a different population of smaller spike‐like deflections (not illustrated), which were taken as sympathetic. In this example, cardiac modulation of these events was not obvious by eye, and the histogram (Fig. 3Ca) shows a relatively low amplitude of cardiac modulation. A similar result was seen for two more of the recordings. However, in two of the other examples (in one animal) cardiac‐related bursts were clear (like those in the distal branch described below) and the relevant histograms showed strong modulation. Sympathetic discharge was not obvious in the remaining recording, but the smallest range of spikes was designated as sympathetic and cardiac modulation observed for this range. In four instances, the gamma ranges gave cardiac modulation (of smaller amplitudes than for their corresponding sympathetic range) and the other two gave noisy histograms (relatively low counts), with weak peaks probably corresponding only to those in their alpha ranges.

### Distal branch

Six recordings from the distal remainder of the internal intercostal nerve (Saywell et al. 2007) were examined, in four cats. Several intercostal and abdominal muscles are innervated by this branch (see Methods), which also has a cutaneous component. Both an inspiratory burst of spikes, assumed to be destined for the parasternal muscle (Taylor 1960; De Troyer et al. 2005), and an expiratory burst were usually present, but the distinction between alpha and gamma recordings was generally less clear than for the lateral branch. In four of these recordings (two in each of two cats), very clear cardiac‐related bursts were visible in the smallest range of spikes. As was typical, in the example shown in Figure 3, the largest of the spike‐like waveforms in the cardiac bursts had amplitudes not much below the level assumed for the alpha/gamma border. The lower level for the gamma range was set a little below the largest events in the cardiac bursts, some overlap between the sympathetic spikes and the gamma spikes being accepted. The histogram in Figure 3Cb shows, not surprisingly, strong cardiac modulation for the sympathetic range. The four recordings with visible cardiac bursts all gave similar strong modulation for the sympathetic range and the other two rather weaker effects. The gamma ranges again showed weaker effects than for the sympathetic range.

### Lumbar (L1) nerves

An ECG signal was obtainable in two cats investigated for L1 discharges, allowing analyses of recordings from 2 nerve branches in each, one innervating internal abdominal oblique muscle and one a more distal remainder (Road et al. 2013). Phasic expiratory alpha and gamma spikes were distinguished for each of these, the distinction being particularly clear for the internal abdominal oblique branch, like the T8 lateral branch in Figure 3. In all four recordings, smaller spikes (presumed sympathetic) were also visible, so three ranges similar to those used for the intercostal nerves were defined for all nerves. For two of the nerves (both in one cat) no modulation was detectable in the histograms for the possible sympathetic level, but the other two both gave clear effects. For the gamma ranges, the two nerves that showed cardiac modulation showed weaker versions, the other two showed none (except for one, an effect similar to its alpha range, but weaker).

### Internal intercostal nerve filaments

**Figure 4:**
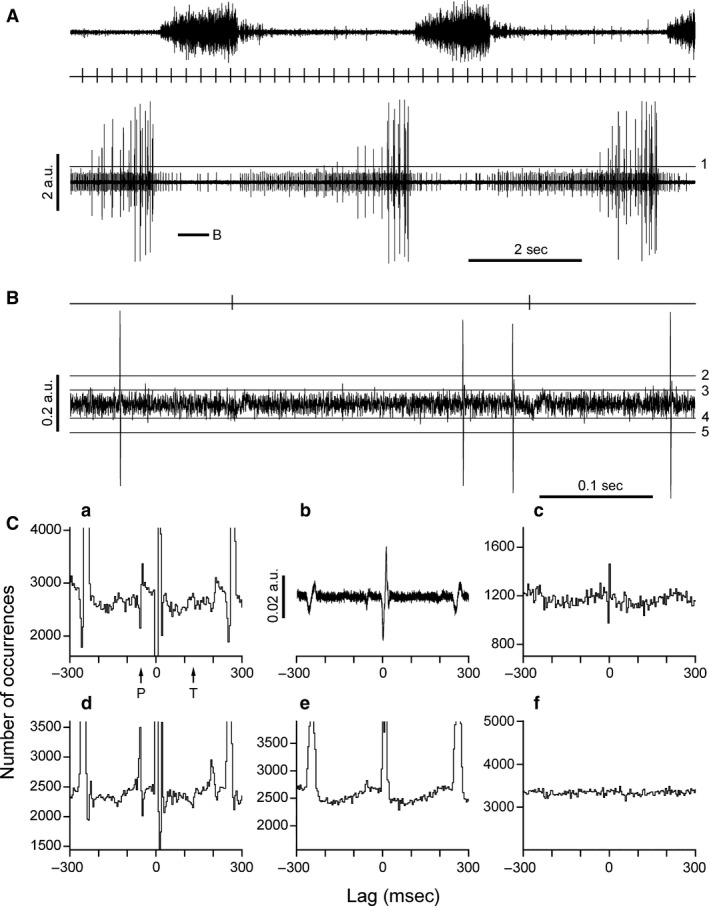
Analysis of a recording where the ECG artifact was appreciable. (A) from above: T6 external intercostal nerve, *R*‐wave events, T8 internal intercostal nerve filament. (B) extract from the filament recording (as indicated below filament trace in A) together with *R*‐wave events (time scale ×20, gain ×10). No cardiac‐related bursts visible in (B), but *R*‐waves are visible. Boundaries for spike amplitude ranges indicated by lines 1–3, as in Figure 1. (C) a, c–e, cross‐correlation histograms from the data of A,B: a, sympathetic range (levels 3–2); c, gamma range (levels 2–1); d, histogram derived from negative‐going spikes (levels 4–5): note that the ECG artifacts in this histogram are sign‐reversed compared to those in a, including components related to both the *P*‐ and *T*‐waves (arrowed). e, average of the histograms in a and d: note that the components for the *P*‐ and *T*‐ waves cancel out almost entirely, revealing the underlying sinusoid‐like modulation. b, *R*‐wave‐triggered average of the filament recording: the *P*‐wave is clear, the *T*‐wave just detectable. Calibration in the same arbitrary units as in (A) and (B). 6696 *R*‐wave events. *R*‐wave period, 254 msec. f, cross‐correlation histogram from the gamma range of the filament recording in Figure 3 (typical for the gamma range of filament recordings). Histogram ordinate ranges, 0.6–1.5 m. Bin widths 4 msec.

These are of particular interest because of the original observations of Sears (1964b), where the efferent spikes were noted as having “two distinct sizes”, neither comprising sympathetic efferents, because they survived section of the gray ramus communicans. The records analyzed here were also composed of spikes of two distinct size ranges (Figs. 3, 4). Further, at the normal recording gain, in contrast to the other recordings described above, it was hard to detect any smaller spikes above the baseline noise. However, when the recordings were examined at higher gain, very small spikes became apparent, including cardiac‐related bursts (Fig. 3B), and were designated as sympathetic (see indicated amplitude ranges). In the histogram for this example, this range gave a clear cardiac modulation, together with an artifactual peak at zero lag, related to the *R*‐wave of the ECG (Fig. 3Cc). The ECG itself was not visible in the raw data from this filament, but was apparent in the *R*‐wave related average (not illustrated). The rather sharp top of the waveform probably also represents an artifact related to the *P*‐wave (see below, this section), but this effect is minor.

Six recordings of filament discharges (including this one) were analyzed. Four (in two cats) were similar to this one, with very small spikes showing cardiac bursts and strong cardiac modulation in the histograms for the sympathetic range. The other two recordings showed very small spikes without visible cardiac bursts (one described below) both giving relatively weak cardiac modulation. For the gamma ranges, five recordings gave flat or almost flat histograms and the other is described below.

Figure 4 shows the analysis of this other filament recording, which is of interest firstly because it is one of the recordings where an ECG waveform was clear in the original recording. At high gain, this may be seen to have an amplitude comparable to that of the very small spikes, which this time did not show obvious cardiac bursts. Nevertheless, cardiac modulation of these very small spikes (see illustrated levels) could be demonstrated in their histogram, though to do this, care was needed to separate rather strong ECG‐related artifacts. The *QRS* artifact is obvious (−4 to +12 msec, truncated in Fig. 4Ca), but there were also artifactual components related to the *P* and the *T* waves (compare latencies of the marked components in the histogram with the *R*‐wave triggered average waveform in Fig. 4Cb). To differentiate the cardiac modulation of the nerve discharges from these two, a second histogram was calculated (Fig. 4Cd), selecting spikes by triggering with the opposite polarity and choosing an amplitude range as near as possibly equivalent to the previous triggers (see levels in Fig. 4B), so as to give approximately the same value of mean counts in the histogram. The biphasic artifact corresponding to the *P*‐wave and the monophasic one relating to the *T*‐wave were reversed in this new histogram, whereas the underlying wide peak was more‐or‐less constant. The process generating these artifacts was reasonably linear, so that when an average of the two histograms (a and d) was constructed (e), the artifacts almost disappeared, leaving a clear representation of the wide peak (plus the *QRS* artifact). Similar discrimination against ECG artifacts was required for the sympathetic range for five of the filament recordings, for 3 of the major internal intercostal nerve branches and for two of the external intercostal nerves.

This recording is also of interest because the histogram for the gamma range here (Fig. 4Cc) showed clear cardiac modulation. In many of the recordings described above, the gamma range may have shown similar modulation, but because it was weaker than for the accompanying modulation for the sympathetic range, it would be easy to hypothesize that the occurrence of gamma spikes was independent of the cardiac cycle and the modulation was present only because of overlapping amplitudes for the spikes of these two ranges. This would be consistent with the more‐or‐less flat histograms for five‐sixth of the filament recordings (e.g. Fig. 4Cf). However, for the filament recording represented in the rest of Figure 4, the gamma spikes were very distinctly discriminated from both the sympathetic and alpha ranges (Fig. 4A and B). Nevertheless, the cardiac modulation for the gamma range (Fig. 4Cc) was of similar amplitude to that for the sympathetic range. It is hard to imagine that this effect for the gamma range could have arisen via what could only have been a low level of contamination from the sympathetic range.

### Quantitative summaries

**Figure 5:**
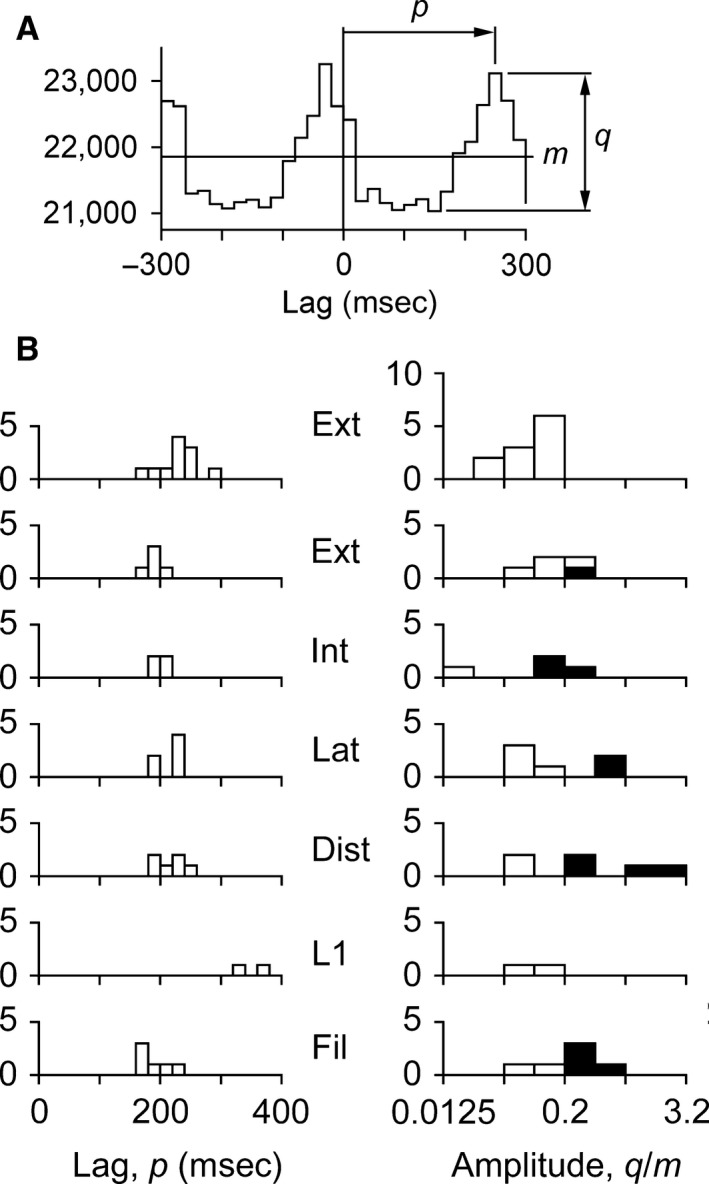
Quantitative summary. (A) example of measurements of lags (*p*) and amplitudes (*q/m*) for the cardiac modulation in the cross‐correlation histograms. *m* is the mean count, calculated over ±0.6 sec. (A) bin‐width of 20 msec was used, (B) Distributions of lags (left) and amplitudes (right), according to the nerves recorded: Ext, external intercostal nerve; Int, whole internal intercostal nerve; Lat, lateral branch of that nerve; Dist, distal branch of that nerve; L1, branches of L1 ventral ramus; Fil, filament of internal intercostal nerve. Two groups of experiments shown separately for Ext: top row, vagotomised; 2nd row, vagi intact. Filled areas, recordings with visible cardiac bursts. Note log scale for amplitude, with a bin‐width equivalent to a factor of 2. Data of Figure 1 used for the example in A.

Lags and amplitudes of the histogram waveforms for the sympathetic ranges were measured using a bin‐width of 20 ms, to give some smoothing of an otherwise noisy signal. An exception to this was made for those like Figure 4, where a by‐eye interpolation on the histograms in 4 msec bins was used instead, so as to maintain discrimination against residual ECG artifacts. Amplitudes were measured trough‐to‐peak during the first cardiac cycle with a positive lag and expressed as a multiple of the mean count (*m*), measured over ±0.6 sec. Lags were measured to the maximum value for that cycle. The procedure is illustrated in Figure 5A and the measurements summarized in Figure 5B. The cardiac cycle times, estimated from the *R*‐wave autocorrelation histograms, ranged from 232 to 330 msec. Lags were relatively consistent, with two exceptions. First, when the two groups of experiments for the external intercostal nerve were considered separately (first two rows in Fig. 5B), the lags for the first group (vagotomised, first row, median 230 msec) tended to be longer than those for the second group (vagi intact, second row, median 190 msec). Second, the lags for the two examples from the lumbar nerves (330 and 360 msec) were the greatest observed. These compare with the mean value (±SD) for the rest of thoracic population of 200 ± 22 msec (*n* = 27, vagi intact preparations only) or with the mean for the thoracic nerves in the same animal (215 msec).

The amplitudes showed considerable variation, 0.02*–*1.45 m (note the log scale for amplitude in Fig. 5B). The recordings where cardiac modulation was visible by eye showed the largest amplitude (filled areas). There were no obvious differences between the values for the different nerves, with the one exception that a difference was detected for the external intercostal nerve for the two different preparations investigated. The amplitudes for the first group (vagotomised, top row in Fig. 5B, median 0.122 m), were smaller than those for the second group (second row, median 0.179 m). The difference was significant for *P* < 0.05, (Mann–Whitney, single‐sided).

**Figure 6:**
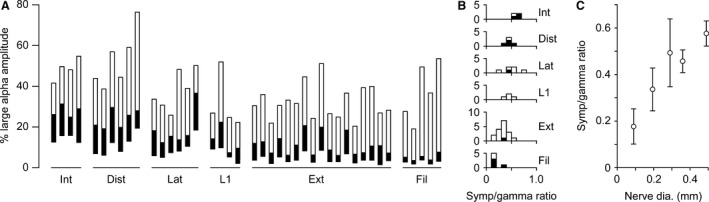
Distributions of the spike amplitude levels used. (A) spike amplitude ranges, expressed in terms of the amplitudes of the largest alpha spikes (see text). Each bar represents one recording: filled area, sympathetic range; open area, gamma range. Nerve designations as in Figure 5, but the plots are arranged in descending order of nerve diameter. (B) distributions of the ratios of the upper limit of sympathetic range to the upper limit of the gamma range. Filled areas, recordings with visible cardiac bursts. (C) values of the ratios shown in B (means ± SD) plotted against nerve diameters measured from Sears (1964a) and/or Duron et al. (1978) (see text). Although it was possible to put the L1 nerves into the order of diameters (from visual assessment of their diameters during the dissections), no histology was available for these branches, so they do not contribute to the plot in C.

During the course of the above analyses, it became obvious that the spike amplitude levels chosen to separate the different efferent groups varied systematically with the nerve categories. These levels are summarized in Figure 6A, measured from the mean of the baseline recording, then expressed as a percentage of the amplitude of the largest alpha spikes. It should be emphasized that, at this stage, this is not strictly an experimental result, but a summary of the values chosen on the basis of subjective judgements, as described above. Also, the values are approximate, since the amplitude of the largest alpha spikes varied considerably from breath to breath and sometimes also more slowly with time. The few individual outlying spikes with especially large amplitudes (most likely arising by superposition) were excluded and we took an approximate by‐eye average to allow for the rest of the variation. For any one nerve category, the three boundary amplitude levels appeared to vary to a great extent in parallel between the different recordings, suggesting an underlying strong effect from the largest alpha spike amplitude. This probably represents variation in recruitment of alpha motoneurones according to anesthetic or CO_2_ levels. Note that the alpha motoneurone discharges in these preparations would generally be considered as strong, on account of the moderate hypercapnia.

It is also obvious that there are differences between the different nerve categories, which have now been arranged in descending order of nerve diameter. One of these has a trivial cause. The thicker nerves must give a smaller spike size, via Ohm's law, so the low level for the sympathetic range (the bottom of each bar in Fig. 6A), which mostly represents discrimination against amplifier noise, appears higher in this plot for the thicker nerves. The more interesting result is that the proportion of total range occupied by the sympathetic range also varies with the nerve category. To escape (at least in part) the influence of the variability in the populations of alpha spikes, we have compared the ratios of the maximum sizes of the spikes in the sympathetic and the gamma ranges across the different nerve categories (Fig. 6B). A clear sequence is present, where the mean ratio for the internal intercostal nerve group is more than 3 times larger than for the internal intercostal nerve filaments. To help reduce the subjectivity involved in this plot, we have indicated the recordings where cardiac bursts were visible in the raw data (filled bars in B), which are the examples which involved the lowest degree of subjectivity in choosing the amplitude level for the gamma/sympathetic border. These examples are nevertheless typical of the rest in this plot. These results were then further quantified by relating the same measurements to measurements of nerve diameter (Fig. 6C). Diameters were measured from the illustrations in Sears (1964a) or, for two instances (whole internal intercostal and external intercostal nerves), using an average value from Sears (1964a) and from Duron et al. (1978).

### Further checks on the appropriateness of the sympathetic amplitude ranges

The relationship in Figure 6C is very clear. Nevertheless, to reduce the subjectivity still further, the cross‐correlation measurements were repeated in selected data, to check how closely the near‐sinusoidal cardiac modulation of the sympathetic range was limited to the particular amplitude ranges chosen. This was done using narrower ranges of spike amplitude, which together covered the previous sympathetic range. Filament recordings were excluded, because the sympathetic spikes were too close to the amplifier noise for such an analysis, but such analysis was in any case unnecessary for these recordings, because a clear separation was obvious between the spikes assigned to the sympathetic or to the gamma ranges. We further excluded: (1) the other recordings like that in Figure 4, where large ECG artifacts were present; (2) a number of other runs where either the activity levels or the shortness of the runs meant that the histograms derived from narrow spike amplitude ranges would have had too few counts and/or where the cardiac modulation was weak, any of which would have made the histograms too noisy. There then remained 17 recordings to be analyzed, 5/17 of the external intercostal nerve recordings, together with 12/20 from the other intercostal or abdominal nerves. A bin‐width of 20 msec was used, as in Figure 5, but to reduce noise further, the individual histograms were smoothed by 3‐point adjacent averaging before measuring their modulation amplitudes.

**Figure 7:**
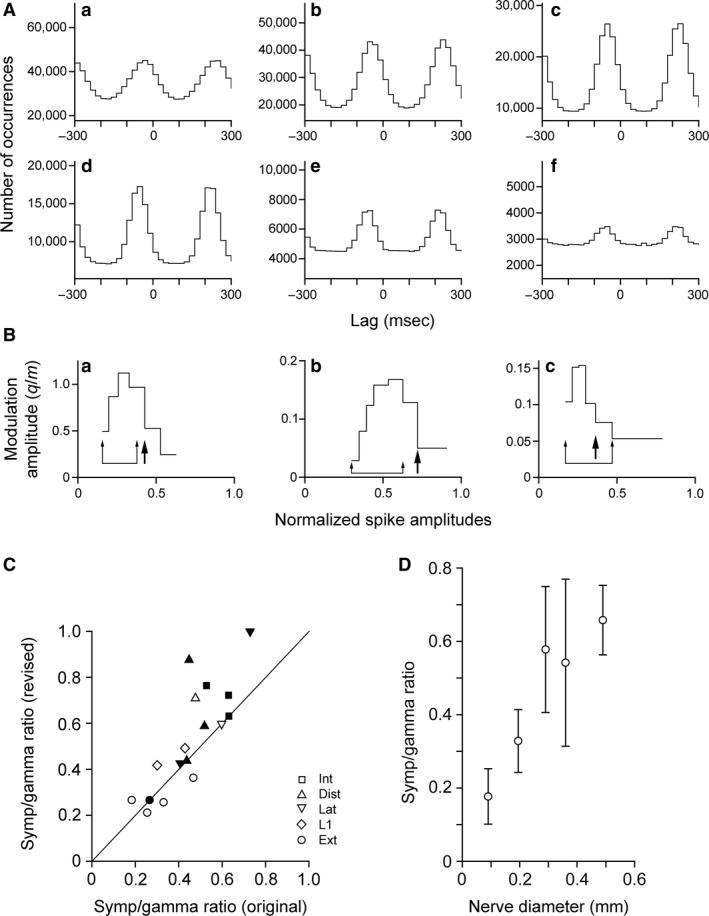
Verification of appropriateness for sympathetic spike amplitude levels. (A) cross‐correlation histograms for narrow spike amplitude ranges spanning the sympathetic range previously used, for the data of Fig. 3 (T8 Dist). Histograms for nonoverlapping ranges are shown, with increasing spike amplitudes going from a to f. Bin‐widths, 20 msec, histograms smoothed by 3‐point adjacent averaging. Histogram ordinate ranges, 0.5–2.0 m. (B): a, plot of modulation amplitude (*q/m*, as in Fig. 5) measured from the series in A (the six bins in this histogram indicate the ranges of amplitudes used for the six cross‐correlation histograms in (A); b, similar plot from the data of Figure 2 (T9L Int); c, similar plot from the data of Figure 1. The abscissae show spike amplitudes normalized to the amplitudes at the alpha/gamma border. The ranges originally assigned as sympathetic are indicated by narrow arrows joined by a line. Revised values for the sympathetic/gamma ratio (see text) were estimated as the spike amplitudes in these plots where the cardiac modulation was reduced to 50% of its maximum value (thick arrows). The spike amplitude ranges needed to be wider for the larger amplitudes in order to obtain enough counts in the histograms to maintain a reasonable signal‐to‐noise ratio, the spike rates being lower for the larger amplitude spikes. (C) revised versus original values for the sympathetic/gamma ratio. Nerve designations (as in Fig. 5) indicated by different symbols. Filled symbols, recordings with visible cardiac bursts. (D) same plot as in Figure 6C, but with revised values for the sympathetic/gamma ratio substituted for the original ones for the 17 recordings represented in B.

Examples of the histograms obtained in this way from one recording (that from the T8 distal branch in Fig. 3) are included in Figure 7A and a plot of the modulation amplitudes, related to the spike amplitude levels used, is shown in Figure 7Ba. The spike amplitudes are normalized by the maximum gamma spike amplitude, that is, by the upper limit of the gamma range. It may be seen that the spike amplitude range where the modulation amplitude is substantial is quite similar to the range originally assigned to be sympathetic (joined thin arrows), though extending to slightly larger spike amplitudes. The same is true for a second example (Fig. 7 Bb, from the T9L recording in Fig. 2), whereas in a third example (Fig. 7Bc, from the external intercostal nerve recording in Fig. 1), the bias is in the opposite direction. Here, the higher modulation amplitudes are also concentrated within the original sympathetic spike amplitude range, but toward the lower end. Not surprisingly, given the previous observations of some cardiac modulation for the gamma range, the modulation was never seen to be zero in these examples. We could not test at higher spike amplitude levels without interference via effects from the (rather different) modulation seen for alpha discharges (Ford and Kirkwood 2018).

The spike amplitude level where the modulation amplitude had fallen to 50% of its maximum (thick arrows in Fig. 7B) was then chosen as an index, so as to make a general comparison of these analyses with the original sympathetic/gamma ratios used in Figure 6. This index, “Symp/gamma ratio (revised)” is plotted in Figure 7C against the original ratios for all 17 of the analyses. For all but 4 of these, the two measures are very close to each other (the line is the line of equality), including examples from all of the nerve categories and including examples both with (filled symbols) and without (open symbols) visible cardiac modulation in the raw data. The other four analyses, all involving high values of the ratio and three of them with visible cardiac modulation in the raw data, showed even higher values for the revised values than for the original assumptions. A resulting re‐plot of Figure 6C, now using the revised values for the 17 reanalyzed examples, but otherwise the original values, is shown in Figure 7D. The standard deviations are higher for the internal intercostal nerves and their major branches than previously, but the overall relationship between the nerve diameter and the sympathetic/gamma ratio is just as clear. The slope is rather steeper.

## Discussion

As far as we know, the direct comparisons here between the discharges of sympathetic and somatic efferent discharges in the same nerves comprise a new result. We need to be certain therefore that the sympathetic discharges are what we claim them to be. First, the general appearance of obvious cardiac bursts in all categories of nerves, are very similar to those reported extensively in the literature. Then, the histograms from those recordings without obvious cardiac bursts were very similar to those with the bursts (for instance the peaks in these two groups had very similar lags and, for each category of nerve, the spikes had similar amplitudes). The analyses in Figure 7 then show that the spike amplitude level used to define the sympathetic discharges were reasonably appropriate (assuming that cardiac modulation is a sufficient identifier), both for the nerves with and the nerves without obvious bursts. Further evidence consistent with our identification of sympathetic discharges is the reduced strength of the modulation for the external intercostal nerves in the first (vagotomised) group of experiments as compared to the second, likely resulting from lower baroreceptor inhibition in the first group, as a consequence of the loss of aortic baroreceptor input. Additional support comes from the measured lags for the histograms from the two abdominal nerves, which were longer than those for the thoracic nerves. About half of the difference, 71 msec, can be ascribed to conduction delays. These were calculated as follows: 50 msec for an extra 50 mm peripheral conduction at 1 msec^‐1^, plus 21 msec for extra central conduction (T8 to L1 being 83 mm in this cat) at 4 msec^‐1^ (McAllen 1986). The remaining difference should then be ascribed to a longer central delay and/or to even slower peripheral conduction if, as was likely, the temperature of the peripheral nerves in question was appreciably lower than the core temperature (Road et al. 2013).

A somewhat (at first sight) surprising result is the range of spike sizes observed. The very small size of spikes seen in the filaments is close to what one might expect for single C‐fibers, as supported by the following calculation. Stein and Pearson (1971), gave a theoretical formula for the amplitude of a spike in an umyelinated fiber as *V(a/b)* ^*2*^, where *V* is the amplitude of the intracellular action potential, *a* is the diameter of the axon and *b* is the diameter of the nerve, this formula being empirically supported by direct measurements (Pearson et al. 1970). For an action potential of 100 mV in a 1 *μ*m fiber in a filament of 0.1 mm, this predicts a spike of 100 × 10^−4^ mV = 10 *μ*V. However, this would be for a nerve consisting of only unmyelinated fibers. Here we may modify this by assuming that the extracellular current will not flow through the myelinated fibers. We measured the proportion of the nerve cross‐sectional area that these occupy from the fiber diameter spectra for the equivalent nerves illustrated in Sears (1964a). For the 4 nerve categories included in Figure 6C (excluding the filament), the average proportional area of these was 66%. The predicted spike amplitude should therefore be increased by a factor of 3, that is to 30 *μ*V. The amplitudes of the gamma spikes in the recordings of Sears (1964b), made under identical conditions to ours, were around 200 *μ*V, meaning that the value for the maximum sympathetic amplitude for filaments here, 0.18 times the gamma amplitude (indicated by the lowest point in Fig. 6C or 7D), corresponds to around 36 *μ*V, close to the prediction.

The apparently much larger spike‐like events in the other nerve recordings, which overlapped in amplitude with the gamma spikes are then most likely to have arisen by superposition of unit spikes. This is well‐recognized to occur for sympathetic discharges. In the very first recordings, Adrian et al. (1932) referred to these discharges as “waves”, assuming a multi‐unit origin. McAllen and Malpas (1997) also pointed this out, writing, “Fortunately, however, their ongoing activity can be measured from whole‐nerve recordings because large numbers of nerve fibers fire action potentials at the same time to give “bursts” or waves of summed spikes”. Other early authors illustrated the phenomenon, a particularly clear example being Taylor and Gebber (1975), where their Figure 1 shows how, for one such single burst, the commonly recorded high‐pass filtered signal, which has the appearance of a burst of spikes, actually represents the filtered noise on the summit of a relatively smooth wave formed from a very much greater number of summed spikes.

This must have been the same in our recordings, although we are now making this assertion with respect to a tonic discharge, rather than a burst (the tonic rate nevertheless modulated by the cardiac and respiratory rhythms). Thus, we are saying that the spike‐like events in Figure 1, whose amplitude and rate decrease during the course of expiration, are not unit spikes but (neural) noise transients made to look like spikes by the filters in our amplifiers. In these terms, the relationship between the relative amplitudes of these events and the nerve diameters can be predicted, as follows. We assume each sympathetic axon action potential gives a unit‐sized current flowing in the extracellular tissue between the electrodes, and that large numbers of these units summate with random occurrence. This summated signal will have Poisson‐like statistics, so the standard deviation of the amplitudes of the resulting transients in the signal may approximate to being proportional to the square root of the mean number of events in any time interval. The amplitudes we chose as representing the maximum for the sympathetic range are most likely to represent a multiple of the standard deviation. Assuming similar firing rates in the different fibers, the “maximum” amplitudes of these current transients will therefore be proportional to the square root of the number of sympathetic fibers. However, the voltage signal created by these current transients will be reduced according to the thickness of the nerve. Assuming now that the proportion of sympathetic fibers is constant in the different nerves means that the extracellular resistance of the nerve between the electrodes will be inversely proportional to the number of these fibers. Thus, the “maximum” voltage amplitude of the sympathetic transients should be inversely proportional to the square root of number of fibers. In contrast, for individual somatic spikes, such as the gamma spikes, their amplitudes will be proportional to the resistance and thus inversely proportional to the number of fibers. Therefore, the ratio of maximum amplitudes of the sympathetic transients and the gamma spikes should be proportional to the square root of the number of fibers. Since the number of fibers should be proportional to the cross‐sectional area of the nerve, then this ratio should be proportional to the square root of the area, i.e. should be directly proportional to the diameter of the nerve. There are several approximations and assumptions in this calculation, but the end result is remarkably similar to what was observed (Figs. 6C and 7D).

The calculations above thus can explain the observations that the sympathetic spike‐like events overlap in size with the gamma spikes. They also add a little more confidence that the activity we have assumed as sympathetic really does have that identity, and they emphasize what an enormous barrage of sympathetic efferent activity must be present in the thoracic nerves. Individual sympathetic efferents fire at rates of no more than 2 sec^‐1^, suggesting that there must be several hundred, if not more, tonically active sympathetic fibers in each nerve, in order to give the summated activity we have observed. This view is supported by the large number of unmyelinated fibers present in the gray ramus communicans for these segments in the cat. Coggeshall et al. (1976) counted about 5000 in each segment. Interestingly, these authors also described the presence of about 20 myelinated fibers, up to 12 *μ*m in diameter, with evidence that these, too, were postsynaptic sympathetic efferents. If these project into the intercostal nerve and are active, they might provide an alternative explanation for the relatively large sympathetic spikes that we observed in the whole internal intercostal nerves. The absence of these spikes in the filaments might then occur simply on the basis of there being a low probability of one of the few large fibers projecting into any one filament. However, it is hard to see how this explanation would predict the graded effect in Figure 6C, so we think that the spike summation explanation remains, at present, the best explanation. It should also be emphasized, following Sears (1964b), that all the above discussion on spike sizes is contingent on the fact that all our recordings were made from intact, undivided nerve branches that contained, we presume, nerve fibers that were (apart from their cut ends) undamaged. This presumption would not apply to teased preparations.

Although supported by our calculations above, our interpretation of the overlap in spike amplitudes between the sympathetic and gamma populations still depends critically on cardiac modulation identifying discharges of a given amplitude as sympathetic. The alternative would be that the firing of the gamma motoneurones themselves shows cardiac modulation. The best evidence against this is the lack of modulation for the gamma discharges in 5/6 of the filaments, the filaments being where the gamma spikes are most clearly separated by spike amplitude. The cardiac modulation seen in the gamma ranges for the other nerves should then be ascribed entirely to effects spreading from the sympathetic range. Actual overlap in spike amplitudes is not actually necessary for this. Assuming that extensive superposition is required to give the larger amplitude sympathetic discharges, then further superposition effects would also inevitably take place between the sympathetic and gamma discharges and would provide the explanation for why the plots in Figure 7B never approached zero. The cardiac modulation observed in one filament recording that was as strong as in the sympathetic range (Fig. 4) would then have to remain unexplained – an outlier.

### Methodological implications

The considerable overlap in amplitudes between the sympathetic and gamma discharges is an important result for any experimenters measuring activity in efferent neurograms, at least for certain sizes of nerves. Efferent neurograms are frequently used in animal experiments as measures of motor outputs, especially where EMG might be impractical or where fictive behavior is to be studied. In such studies the possibility of gamma, as much as alpha motoneurone activity being involved should always be considered, although this is frequently ignored. The present result, with the amplitude of the sympathetic discharges often being larger than 50% of that of the gamma spikes, means that this third category of efferent activity should now additionally be considered. The recordings where this would be of most concern are those where normal activity has been eliminated or reduced by procedures such as experimental injury, and then restoration of function (often modest) is being sought.

For example, one result that might need some re‐interpretation featured in a recent publication from our own laboratory (de Almeida and Kirkwood 2013), where the strength of cross‐correlations between the spike trains of bulbospinal neurones and motoneurone efferent discharges in the rat was found to be strongly dependent on the efferent spike size. It was suggested in that paper that part of the explanation could be an overlap in spike sizes, consequent on the known overlap in alpha and gamma conduction velocities in this species. One would now need to suggest that, for the lowest part of the range, the effect could also involve an overlap with sympathetic discharges. The assumption in either case is that the connections represented in the cross‐correlations were made mainly to alpha motoneurones, and that a proportion of discharges from the other categories of efferents within the supposed alpha ranges had a dilution effect in the cross‐correlations.

If, on the other hand, one chose to interpret our observations as resulting from a lower degree of overlap between the discharges, but from the occurrence of cardiac modulation in gamma motoneurones, then yet more ambiguities could arise. Care would then be needed to exclude gamma motoneurone spikes in measurements of cardiac modulation of sympathetic discharges, including in human microneurography experiments.

### Functional issues

Generally, we have used the similarity between the sympathetic discharges described here and others reported in the literature to help in the identification of the discharges here as sympathetic. Nevertheless, one difference is apparent: our histograms suggest weaker cardiac modulation than is commonly described for muscle nerves. To put this into context, when our results are compared to other studies using a similar method (cross‐correlation of *R*‐wave with sympathetic spikes or spike‐like events), our strongest examples of cardiac modulation were similar in strength to those illustrated as typical results in previous studies (Jänig et al. 1983; Sato and Schaible 1987; Fatouleh and Macefield 2013). As pointed out by McAllen and Malpas (1997), there are different methods available to quantify sympathetic discharges each with its own advantage. Here, a particular advantage of using whole nerve recordings is that we have avoided the possible selectivity involved either in preparing teased peripheral nerves or in selecting fascicles for recording by microneurography. Such selectivity could create a bias toward the most phasically active fascicles. Indeed, such bias is often explicit (e.g. Boulton et al. 2016). A disadvantage of our method is that we cannot necessarily exclude the presence of gammas as contributing to the apparent tonic background. This is quite likely for records such as those in Figure 2, where for the whole internal intercostal nerves, the overlap in amplitudes between the two populations of spikes was deemed to be high. However, this is likely to be much less so for the large majority of the external intercostal nerves. In the example of Figure 1, during most of expiration (a high proportion of each respiratory cycle), we think the gamma spikes are largely absent, yet, as was generally the case for the external intercostal nerves, the cardiac modulation in sympathetic discharges was relatively modest and could not be seen in the raw recordings.

Assuming this is a real result, it is worth asking whether it is a consequence of something particular to the conditions of our experiments or to the intercostal (and abdominal) nerves. One possibility is our use of an elevated level of CO_2_. This is most often regarded as promoting stronger sympathetic output (Millar and Biscoe 1966; Gregor and Jänig 1977; Priess and Polosa 1977), but this doesn't help the particular question. Another possibility is that cutaneous sympathetic fibers show much less cardiac modulation than do those in muscle nerves (Jänig et al. 1983; Fatouleh and Macefield 2013), so one might suggest that the modest level of modulation in our recordings was the result of recording from mixed nerves. However, this is unlikely to be the reason, because the recordings from the external intercostal nerve, a pure muscle nerve, comprised the group with, if anything, the lowest level of cardiac modulation, even discounting the effect of vagotomy. One might then also ask why the mixed nerves (internal intercostal and its branches) did not show even weaker cardiac modulation. This could be the result of the inhibitory influence of hypercapnia on cutaneous vasoconstrictor fibers (Gregor and Jänig 1977). A third overall possibility might be that the muscles innervated by the recorded nerves were more‐or‐less continuously, even if fictively, active. Muscle nerve sympathetic discharges have been reported to either decrease (Wallin et al. 1992) or increase (Boulton et al. 2014, 2016) with centrally commanded muscle activation, but in all the studies so far, tonic sympathetic activity has been excluded because of the difficulty in microneurographic recordings to distinguish changes in tonic activity of sympathetic efferents, if present, from the undoubted changes in tonic afferent or somatic efferent discharges or from artifactual changes resulting from electrode movement. The tonic background that we suggest was present in our sympathetic discharges may therefore have occurred specifically as an accompaniment to somatic efferent activity. A number of authors have suggested possible functional advantages in synaptic transmission for sympathetic efferents to fire in bursts (e.g. McAllen & Malpas, 1997). We do not need to reiterate these arguments here, but merely emphasize that, for some situations, such as the conditions of our experiments, perhaps considerations of firing in bursts should not be the whole story, and that the functional effects of a considerable tonic background should not be ignored.

## Conflict of Interest

No conflicts of interest exist with respect to this report.
